# Positive and Negative Regulation of Gli Activity by Kif7 in the Zebrafish Embryo

**DOI:** 10.1371/journal.pgen.1003955

**Published:** 2013-12-05

**Authors:** Ashish Kumar Maurya, Jin Ben, Zhonghua Zhao, Raymond Teck Ho Lee, Weixin Niah, Ashley Shu Mei Ng, Audrey Iyu, Weimiao Yu, Stone Elworthy, Fredericus J. M. van Eeden, Philip William Ingham

**Affiliations:** 1 A*STAR Institute of Molecular & Cell Biology, Proteos, Singapore; 2 Department of Biological Sciences, National University of Singapore, Singapore; 3 MRC Centre for Developmental and Biomedical Genetics, University of Sheffield, Western Bank, Sheffield, United Kingdom; University of Pennsylvania School of Medicine, United States of America

## Abstract

Loss of function mutations of Kif7, the vertebrate orthologue of the *Drosophila* Hh pathway component Costal2, cause defects in the limbs and neural tubes of mice, attributable to ectopic expression of Hh target genes. While this implies a functional conservation of Cos2 and Kif7 between flies and vertebrates, the association of Kif7 with the primary cilium, an organelle absent from most *Drosophila* cells, suggests their mechanisms of action may have diverged. Here, using mutant alleles induced by Zinc Finger Nuclease-mediated targeted mutagenesis, we show that in zebrafish, Kif7 acts principally to suppress the activity of the Gli1 transcription factor. Notably, we find that endogenous Kif7 protein accumulates not only in the primary cilium, as previously observed in mammalian cells, but also in cytoplasmic puncta that disperse in response to Hh pathway activation. Moreover, we show that *Drosophila* Costal2 can substitute for Kif7, suggesting a conserved mode of action of the two proteins. We show that Kif7 interacts with both Gli1 and Gli2a and suggest that it functions to sequester Gli proteins in the cytoplasm, in a manner analogous to the regulation of Ci by Cos2 in *Drosophila*. We also show that zebrafish Kif7 potentiates Gli2a activity by promoting its dissociation from the Suppressor of Fused (Sufu) protein and present evidence that it mediates a Smo dependent modification of the full length form of Gli2a. Surprisingly, the function of Kif7 in the zebrafish embryo appears restricted principally to mesodermal derivatives, its inactivation having little effect on neural tube patterning, even when Sufu protein levels are depleted. Remarkably, zebrafish lacking all Kif7 function are viable, in contrast to the peri-natal lethality of mouse *kif7* mutants but similar to some Acrocallosal or Joubert syndrome patients who are homozygous for loss of function *KIF7* alleles.

## Author Summary

Hedgehog (Hh) proteins activate one of a handful of signaling pathways that orchestrate the development of animal embryos, controlling cell type specification, proliferation and survival in a variety of contexts. In *Drosophila*, the Cos2 protein plays a key role in modulating the response of cells to Hh signaling, while mutant forms of its human counterpart KIF7 are associated with a class of developmental defects known as ciliopathies. Studies in mouse have implied that Kif7 functions principally in the primary cilium, an organelle required for Hh signaling in vertebrates but absent from most *Drosophila* cells, suggesting a divergence in the mechanisms of action between phyla. Here we describe the generation of *kif7* mutations in the zebrafish as well as the first analysis of endogenous Kif7 protein distribution in a vertebrate embryo. We find that Kif7 acts principally to restrain Gli1 activity and suggest that it functions to sequester the Gli transcription factors, similar to its *Drosophila* counterpart Cos2, which we also show can partially substitute for Kif7 function in the zebrafish embryo. Consistent with this model we show that Kif7 protein accumulates both in the primary cilia and in cytoplasmic puncta from which it disperses in response to Hh pathway activation.

## Introduction

Hedgehog (Hh) proteins play a fundamental role in animal development, controlling cell type specification, proliferation and survival in a variety of contexts through a signaling pathway, the core components of which are shared across species [reviewed in 1], [2], [3]. Hh signaling also functions post-embryonically, regulating tissue homeostasis [4], metabolism [5] and physiological processes [6], while aberrant pathway activity underlies the etiology of a variety of cancers [7], [8].

The kinesin family protein Costal2 (Cos2) is a central component of the intracellular Hedgehog signaling complex in *Drosophila* [9]. Cos2 physically interacts with the Gli family protein Cubitus interruptus (Ci), restraining the transcriptional activating activity of its full length form both by anchoring it in the cytoplasm as well as by recruiting the serine-threonine kinases that prime it for processing into a truncated transcriptional repressor [10], [11], [12]. Consistent with these effects, loss of Cos2 activity results in the ectopic activation of Hh target genes, both in embryos and imaginal discs [13], [14]. In addition to its negative regulatory role, Cos2 has also been shown to potentiate Hh pathway activity by promoting the dissociation of Ci from Suppressor of Fused (Sufu) [15], another negative pathway regulator that acts to inhibit nuclear import of Ci [10], [16].

The closest vertebrate homologue of Cos2 is Kif7 [17], mouse mutants of which similarly exhibit evidence both of gain and loss of Hh pathway activity [18], [19], [20], implying a conservation of Cos2/Kif7 function between insects and vertebrates. Like Cos2, Kif7 promotes the processing of Gli transcription factors to their repressor forms both in mouse and human cells [18], [19], [20], [21] a function that, in part, can explain the Hh gain of function phenotypes in the limbs and in the neural tube of *kif7* mutants. One significant difference between Kif7 and Cos2 however, is the association of the former with the Primary Cilium, an organelle that is absent from most *Drosophila* cells but of central importance for Hh signaling in vertebrates [22], [23]. Tagged forms of Kif7 have been shown to localize to the primary cilium tip in response to Hh pathway activation when expressed in cultured mammalian cells [19], [20]. Similarly, translocation of Gli proteins to the primary cilium tip is also induced by Hh signaling [24], a process that is proposed to be required for their activation through dissociation from Suppressor of Fused (Sufu) [25], [26]. Paradoxically, given its role as a negative regulator of the pathway, loss of Kif7 function abrogates the Hh induced translocation of Gli to the primary cilium tip [19]. Whilst such an effect can be reconciled with the partial attenuation of pathway activation observed in the neural tube of *kif7* mutant embryos [18], [19], [20], how the localization of Kif7 to the primary cilium relates to its repressive function remains unclear.

The first evidence of a conserved function for Kif7 in vertebrate Hh signaling was based on morpholino mediated transient knock-down experiments in zebrafish, [17]. Morphant *kif7*embryos exhibit rather subtle defects in cell fate specification principally in the myotome that contrasts with the more robust de-repression of the Hh response seen in *Drosophila* c*os2* mutants. While this could reflect a divergence in Kif7 function between species, it might also be attributable to the transient nature of the morpholino mediated knock-down. To explore the role of zebrafish Kif7 further, we have generated loss of function alleles by zinc finger nuclease (ZFN) mediated targeted mutagenesis [27], [28], [29] and used these to dissect the role of Kif7 in modulating the activity of the Gli transcription factors. In addition, using an antibody raised against the zebrafish protein, we have analyzed the levels and sub-cellular distribution of endogenous Kif7 in the presence and absence of Hh pathway activation. Our findings imply a previously unrecognized role for Kif7 in sequestering Gli1 in the cytoplasm, a function analogous to that of *Drosophila* Cos2, and suggest that Kif7 functions in the primary cilium principally to potentiate Gli2 activity.

## Results

### Generation of mutant alleles of zebrafish *kif7* using zinc finger nucleases

**Figure 1:**
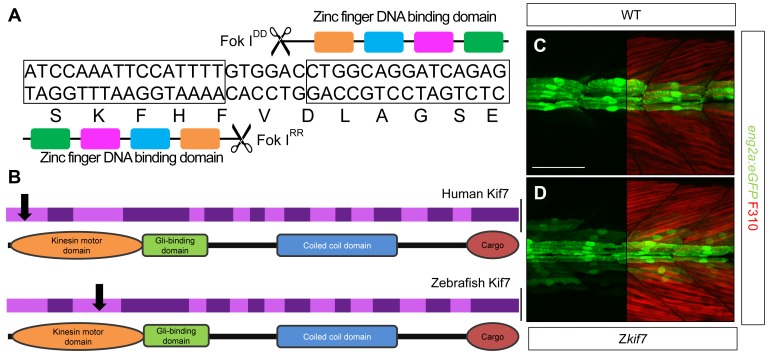
Targeted mutation of the zebrafish *kif7* gene. (A) Schematic representation of the nucleotide sequence in exon 3 of the zebrafish *kif7* gene targeted by the Zinc finger nucleases. (B) Schematic representation of the human and zebrafish Kif7 coding sequences showing conserved exonic structure (dark shading) corresponding to different protein domains (drawn to scale). The black arrows indicate the approximate position of homozygous viable mutations found in some human patients and of the induced lesions in the zebrafish gene. (C,D) Expression of *eng2a:gfp* reporter gene in muscle fibers in the tail somites of wild-type (C) and *kif7* homozygous (D) embryos at 2.5 dpf. Low level ectopic expression of the reporter is detected in fibers surrounding the muscle pioneers in the *kif7* mutants. Merged images showing fast twitch muscle fibers stained with mAb F310 (red) are shown in the right-hand panels. Scale bar: 50 µm.

To analyze the function of zebrafish Kif7, we generated stable germ line transmissible mutant alleles of *kif7* using zinc finger nuclease (ZFN) mediated targeted mutagenesis [27], [28]. A budding yeast-based system identified sequences in the *kif7*coding region that are potentially amenable to targeted mutagenesis. Targeting this sequence in the 3rd coding exon had the potential to create mutations in a region conserved with the mammalian Kif7 protein at cds nt729–765 (A729TCCAAATTCCATTTTGTGGACCTGGCAGGATCAGAG765) (Fig. 1A). Embryos injected with capped RNA encoding a ZFN pair selected for recognition of this sequence were found to carry a variety of deletions or insertions at the target site (data not shown) confirming the efficacy of this approach. Accordingly, we grew up adults from injected embryos and screened their progeny for transmission of *kif7* lesions. Three individuals transmitting deletion mutations were recovered and two alleles that cause frame-shifts resulting in premature termination codons (Table 1) predicted to yield proteins truncated in the motor domain (Fig. 1B) were selected for further analysis.

**Table 1:** Alleles and translational products of zebrafish *kif7* mutants generated with CompoZ ZFN.

Alleles	Genotypes and encoded Kif7 proteins
Wild-type	ATCCAAATTCCATTTTGTGGACCTGGCAGGATCAGAG
	S_244_ K F H F V D L A G S E
*kif7* ^i271^ (8 bp-del: D250RfsX7)	ATCCAAATTCCATTTTGTCAGGATCAGAGCGCATCCT**TAA**
	S_244_ K F H F V ***R I R A H P*** *
*kif7* ^i272^ (7 bp-del: F248SfsX59)	ATCCAAATTCCATTCCTGGCAGGATCAGAGCGCATCCTTAAAACCGGCAACACCGGCGAACGGCTCAAG
	S_244_ K F H ***S W Q D Q S A S L K P A T P A N G S R***
	GAGAGCATTCAGATCAACAGTGGACTTCTTGTTCTTGGAAATGTCATTGGAGCGCTTGGGGACCCCAAA
	***R A F R S T V D F L F L E M S L E R L G T P K***
	AGAAAAGGCACCCATATCCCATACAGGGATTCAAAAATCACCAGGATCT**TAA**
	E K A P I S H T G I Q K S P G S *
*kif7* ^i273^ (3 bp-del: D250del)	ATCCAAATTCCATTTTGTCCTGGCAGGATCAGAG
	S_244_ K F H F V L A G S E

### Loss of zygotic *kif7* function causes a mild Hh pathway de-repression phenotype

Animals homozygous or trans-heterozygous for the *kif7* mutant alleles predicted to encode truncated proteins, completed embryogenesis and showed no defects in the specification of slow-twitch muscle fibres, muscle pioneers (MPs) or medial fast fibres (MFFs) as visualized by Prox1 and Eng2a expression respectively (data not shown), sensitive read-outs of Hh activity in the zebrafish embryo [30]; nor did they display any other manifestations of aberrant Hh pathway activity at 24 hpf. By 2.5 dpf, however, some mutant larvae exhibited ectopic expression of the *eng2a:eGFP* reporter gene in the myotome (Fig. 1C,D). Nevertheless, the homozygous and trans-heterozygous fish were fully viable and grew into phenotypically normal adults. This contrasts with the peri-natal lethality of mice homozygous for *kif7* loss of function alleles [18], [19], [20], but mirrors the finding that some Joubert syndrome patients are homozygous for mutated *KIF7* alleles that cause severe truncation of the protein ([31]; see Fig. 1B).

### Loss of zygotic and maternally derived Kif7 activity causes strong de-repression of Hh signaling in mesodermal derivatives

**Figure 2:**
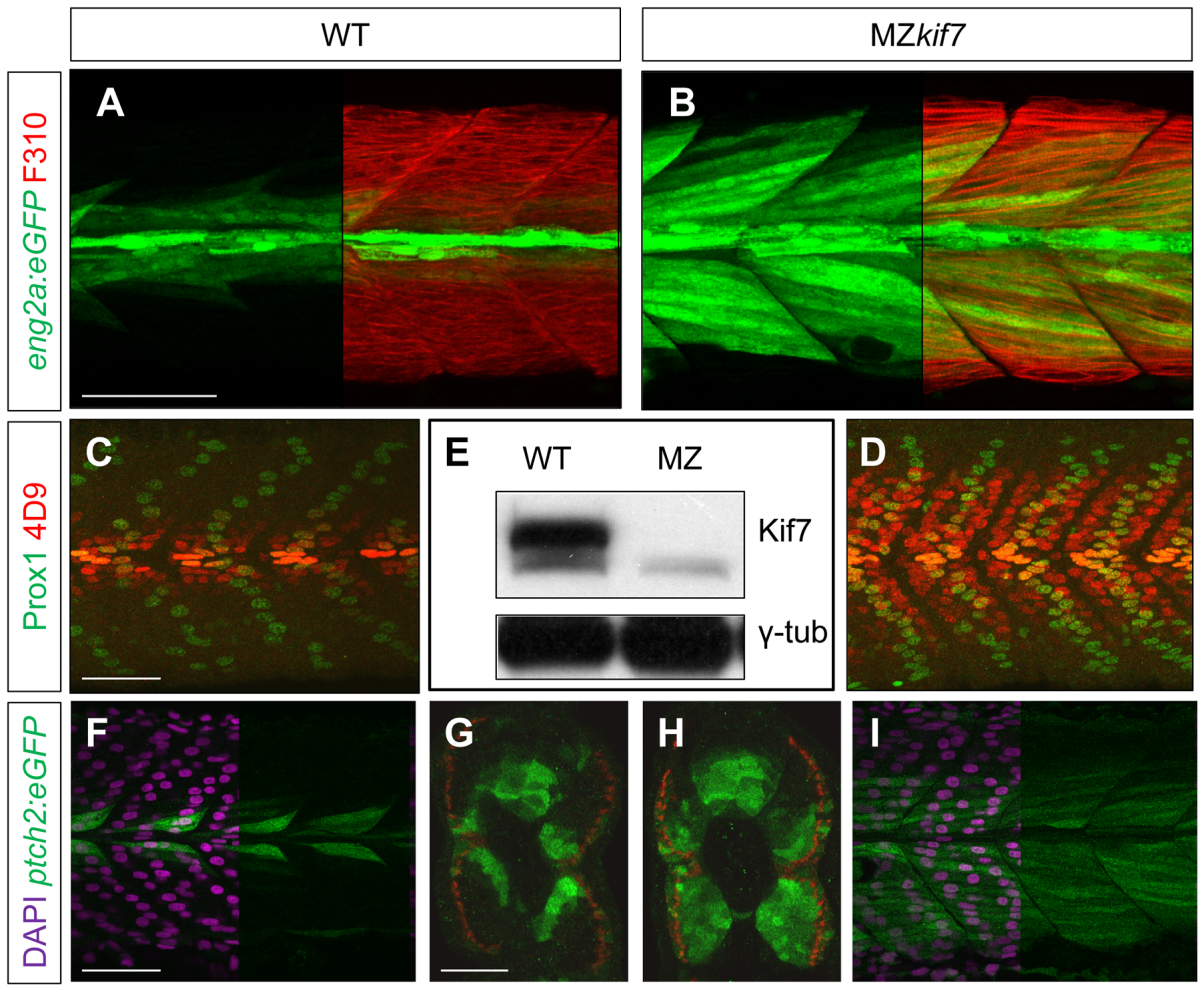
Absence of Kif7 protein and de-repression of Hh target genes in MZ*kif7* embryos. (A,B) Lateral images of 30 hpf wild-type (A) and MZ*kif7* mutant (B) embryos expressing *eng2a:eGFP* (green); note dramatic expansion of *eng2a:eGFP* expression within the fast-twitch fibers revealed by F310 staining (red) in the merged image (right panel). (C,D) Parasagittal optical sections of wild-type (C) and MZ*kif7* mutant (D) 30 hpf embryos stained with anti-Prox1 (green) and mAb4D9 (red) (E) Western blot of wild-type (WT) and MZ*kif7* mutant (MZ) embryo extracts probed with polyclonal rabbit anti-Kif7 antiserum showing complete loss of Kif7 protein (upper band) from MZ*kif7* embryos. Loading control: γ-tubulin (γ-tub). (F,I) Parasagittal and (G,H) transverse optical sections of 30 hpf wild-type (F,G) and MZ*kif7* (H,I) embryos expressing a *ptch2:eGFP* transgene (green) showing the expansion of the *ptch2* expression domain in the myotome and neural tube in the absence of Kif7. Nuclei are revealed in left half (of panels F,I) by DAPI staining (purple). The edge of the myotome is shown by mAbF9 (in G,H) marking the superficial slow-twitch muscle fibers. Scale bar: 50 µm.

**Figure 3:**
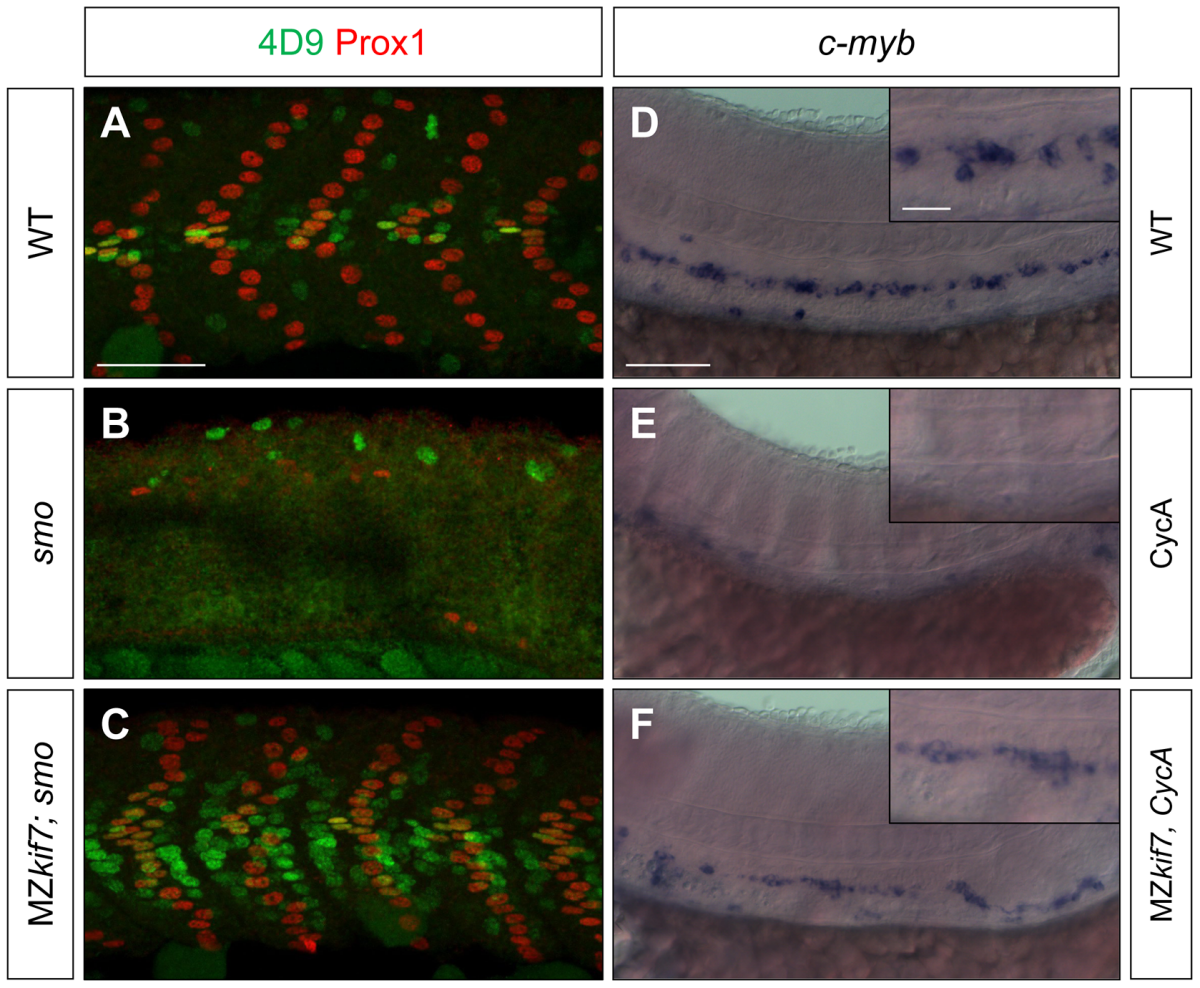
Kif7 acts downstream of Smo to control Hh target genes. (A,B,C) Parasagittal optical sections of 30 hpf wild-type (A), *smo* mutant (B) and *smo*;MZ*kif7* (C) double mutant embryos stained with mAb4D9 (green) and Prox1 (red). Scale bar 50 µm. (D,E,F) Lateral view at the level of the yolk extension of 36 hpf wild-type (D), cyclopamine exposed (E) and MZ*kif7*; cyclopamine (CycA) exposed (F) hybridized with a probe for *c-myb* RNA, marking hematopoietic stem cells in the ventral floor of the dorsal aorta. Scale bar 50 µm (detail in insets; scale bar 10 µm).

**Figure 7:**
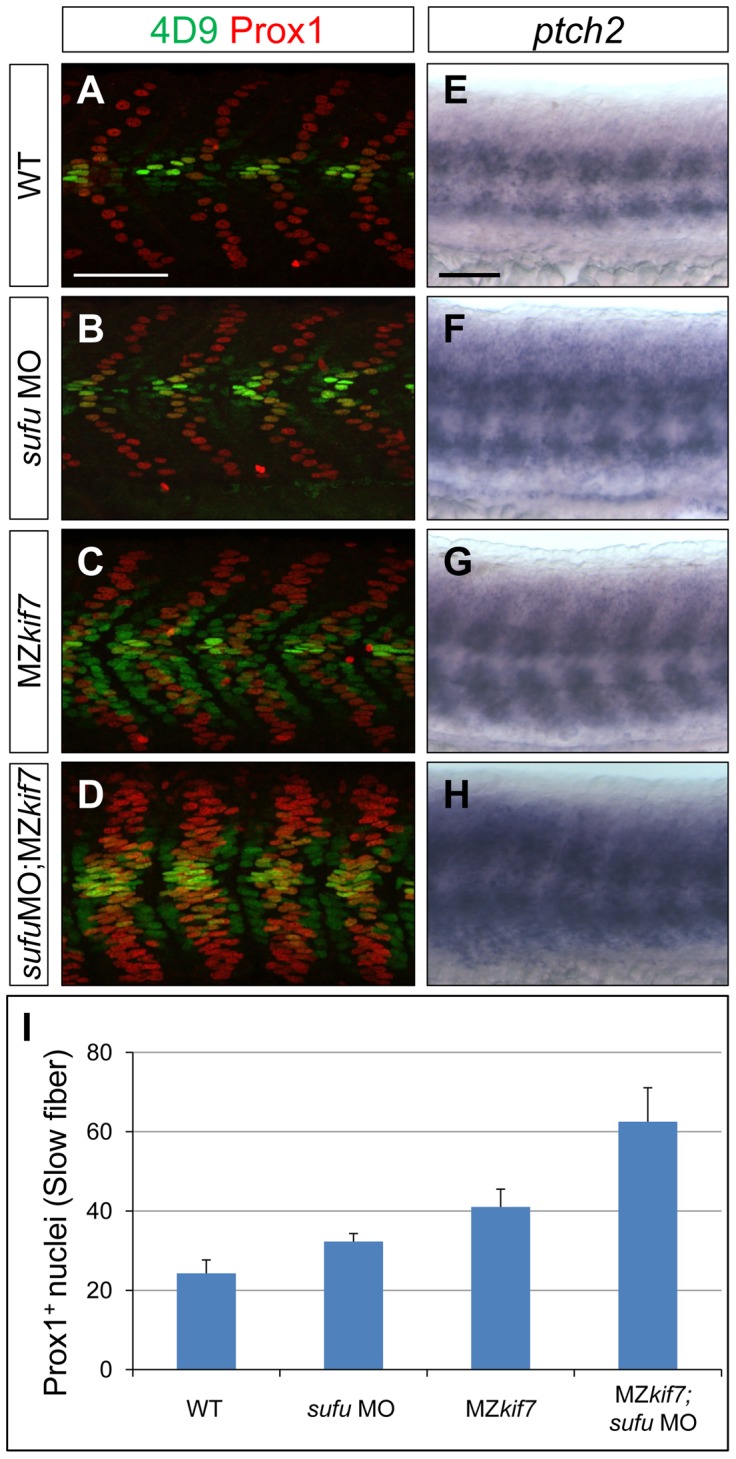
Loss of Sufu further enhances Gli activity in MZ*kif7* mutants. (A–D) Parasagittal optical sections of 30 hpf WT (A,B) or MZ*kif7*(C,D) embryos stained with anti-Prox1 (red) and 4D9 (green) antibodies; morpholino mediated knock-down of *sufu* results in significant increase in Prox1 expressing slow twitch muscle cells in WT (B, quantified in I) and a further increase in MZ*kif7*(D) mutants; Scale bar: 50 µm. (E–H) Lateral view of 30 hpf WT (A) or MZ*kif7*(C) embryos hybridized with an antisense probe for *ptch2* mRNA; MZkif7 embryo shows an expansion of *ptch2* expression; embryos of similar stage and genotype injected with *sufu* MO result in an enhancement of the levels of *ptch2* staining in WT (F) and in MZ*kif7* (H) embryos; Scale bar: 50 µm. (I) Quantification of Prox1^+^ slow twitch muscle fiber nuclei per hemisegment in WT, *sufu* MO injected, MZ*kif7* and MZ*kif7* injected with *sufu* MO. Error bars represent standard deviation obtained from 16–20 hemisegments from >6 embryos. Note the significant increase in MZ*kif7* compared to WT and a further enhancement in slow fibers by removal of Sufu in MZ*kif7* mutants.

We surmised that the lack of disruption of Hh pathway activity in the absence of zygotic *kif7* function could be due to maternally derived *kif7* mRNA present in newly fertilized eggs. To test this inference, we crossed *kif7* homozygous females to *kif7* homozygous males. The resulting maternal and zygotic (MZ) *kif7* mutant embryos were devoid of full length Kif7 protein detectable by Western blot analysis (Fig. 2E) and exhibited a significant expansion of *eng2a:eGFP* reporter gene expression in the myotome at 30 hpf (Fig. 2A,B). Consistent with the de-repression of the Hh pathway implied by this effect, a reporter gene for *ptch2*, a direct target of the Hh pathway, was ectopically expressed throughout the myotomal compartment of the somites (Fig. 2F–I). MZ*kif7* embryos also showed an increase in the number of Prox1^+ve^ slow-twitch muscle cells, the specification of which is Hh–dependent (Fig. 2 C,D; see also Fig. 7I); however, the ectopic expression of Eng, revealed both by the *eng2a:GFP* reporter (Fig. 2B) and by 4D9 mAb staining (Fig. 2D) was restricted to fast-twitch muscle fibers, indicating that loss of Kif7 activity is not sufficient for the maximal pathway activation required for MP induction [30]. Inhibition of Smo activity in MZ*kif7* embryos (via mutation or treatment with the Smo antagonist cyclopamine) had little effect on the ectopic expression of *ptch2* (data not shown) or Eng (Fig. 3C) consistent with Kif7 acting downstream of Smo to suppress transcriptional activation of Hh targets by the Gli transcription factors in the myotome. In both instances, however, there was a loss of MPs, indicating that removal of Kif7 is not sufficient for maximal pathway activation in the absence of Smo (Fig. 3B,C). Hh signaling is also required for definitive haematopoiesis in the zebrafish embryo [32] and inhibition of Smo activity by cyclopamine treatment blocks the formation of haematopoietic stem cells (HSCs) as revealed by the loss of *c-myb* expression in the dorsal aorta (Fig. 3E). Complete elimination of Kif7 activity reversed this effect (Fig. 3F) indicating that Kif7 also acts in the non-myogenic mesoderm to modulate Hh pathway activity.

Loss of Kif7 has previously been reported to disrupt left-right (L-R) asymmetry reflecting a presumed role in ciliogensis in Kupfer's vesicle [33]. We analyzed the establishment of L-R asymmetry in MZ*kif7* embryos using *lefty2* expression [34] as an assay and found no example of *situs inversus* (Fig. S1). We conclude that the reported defect is likely an off-target effect of the morpholino.

### Gli2a processing is delayed in the absence of Kif7

**Figure 4:**
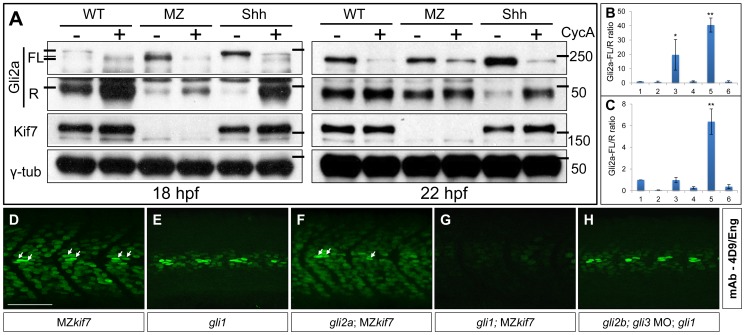
Regulation of Gli processing and activity by Kif7. (A) Western blot analysis showing Gli2a processing in wild-type (WT), MZ*kif7* (MZ) and Shh mRNA injected (Shh) embryos at 18 hpf (left panel) and at 22 hpf (right panel) compared to the same set treated with cyclopamine (CycA). Full-length (FL) and repressor (R) forms of Gli2a are indicated. The lower panels shows the same blot re-probed with Kif7 and γ-tubulin (loading control) antibodies (B,C) Quantification of the Gli2a FL∶R ratio normalized to WT at 18 hpf (B) and 22 hpf (C). Bar graphs numbered 1–6 represent lanes on the blots in (A) from left to right. Error bars represent standard deviation obtained from three independent Western blots including those shown in (A). Single asterisk: P<0.02; double asterisk: P<0.001. (D–H) Parasagittal optical sections of 30 hpf embryos of different genotypes showing expression of Eng-expressing muscle cells as revealed by mAb4D9 (green). The MZ*kif7* phenotype (D) is largely unaffected by removal of Gli2a (F), other than the loss of the some MP cells (arrows); by contrast, Eng expression is nearly eliminated in the MZ*kif7*;*gli1* double mutant embryo (G), despite Gli1 being dispensable for Eng expression in the presence of Kif7 function (E). Knockdown of *gli2b* and *gli3* activity using morpholinos in *gli1* mutants (H) has no effect on Eng expression in the myotome. Scale bar: 50 µm.

Cos2/Kif7 has been implicated in the control of Gli protein processing both in flies and mammals; in the case of *Drosophila*, at least, this is mediated through the recruitment of the kinases that prime the Gli–family protein, Ci, for proteasomal cleavage [12]. Previously we showed that Gli2a is processed in a Hh dependent manner in zebrafish embryos, the ratio of full length (FL) to the truncated repressor (R) form of Gli2a being markedly increased in *ptch1;ptch2* double mutant embryos and substantially decreased in embryos treated with cyclopamine [35]. We investigated the role of Kif7 in Gli2a processing using Western blot analysis to compare the levels of FL and R forms in MZ*kif7* embryos with those in wild-type embryos in which the Hh pathway was fully activated (by injection of Shh mRNA) or fully repressed (by exposure to cyclopamine). At 22 hpf, whereas the FL∶R ratio was significantly increased in the Shh mRNA injected embryos and reduced in embryos exposed to cyclopamine, the ratio did not differ significantly between MZ*kif7* and wild-type embryos, although the levels of FL protein appeared less sensitive to cyclopamine treatment in the MZ*kif7* embryos (Fig. 4A,B). In younger (18 hpf) embryos, by contrast, we observed a consistent increase in the FL∶R ratio in MZ*kif7* embryos, though not as great as in Shh mRNA injected embryos (Fig. 4A,C). At this stage, the FL form of Gli2a appears as a doublet, the slower mobility form of which is sensitive to cyclopamine treatment and therefore presumably Smo dependent. Whereas in Shh mRNA injected embryos, it is this putative Smo dependent form that accumulates at the expense of the truncated R form, in MZ*kif7* it is the cyclopamine resistant form that accumulates (see Fig. 4A). This suggests that Kif7 is required for a Smo-dependent modification of full length Gli2a as well as for its efficient cleavage to the R form. Notably, the increased FL∶R ratio in MZ*kif7* embryos is effectively suppressed by cyclopamine treatment (Fig. 4A,C), suggesting that Kif7 may potentiate Gli2a cleavage by opposing Smo activity.

### Kif7 functions principally to restrain Gli1 activity

Previous studies have shown that Gli1 and Gli2a act redundantly to mediate Hh activity in the myotome [30]; embryos homozygous for loss of function alleles of *gli1* (also known as *detour* (*dtr*): [36]) or *gli2a* [37] show normal specification of Hh-dependent muscle cell types, whereas in the absence of both genes, all Hh-dependent muscle cell types fail to form [30], [37]. By contrast, simultaneous morpholino mediated knockdown of *gli3* and of the *gli2a* paralogue *gli2b* had no discernible effect on Eng expression either in wild-type or *gli1* mutant embryos (data not shown and Fig. 4G). It follows that Gli1 and Gli2a are the principal mediators of Hh signaling in the myotome and that either protein can respond to Hh activity to elicit the full range of cellular responses to the signal. To investigate the role of Kif7 in regulating Gli activity, we generated MZ*kif7* embryos homozygous for either *gli1* or *gli2a* loss of function mutations. MZ*kif7;gli2a* double mutants exhibited little modification of the ectopic Eng expression seen in MZ*kif7* mutants alone, except for a slight reduction in the number of MP cells in each somite (Fig. 4E). By contrast, in MZ*kif7*; *gli1* double mutants, Eng^+ve^ cells were barely discernible in the myotome (Fig. 4F). These findings imply that Kif7 acts principally to restrain Gli1 activity.

### Endogenous Kif7 interacts with Gli1 protein in the developing embryo

**Figure 5:**
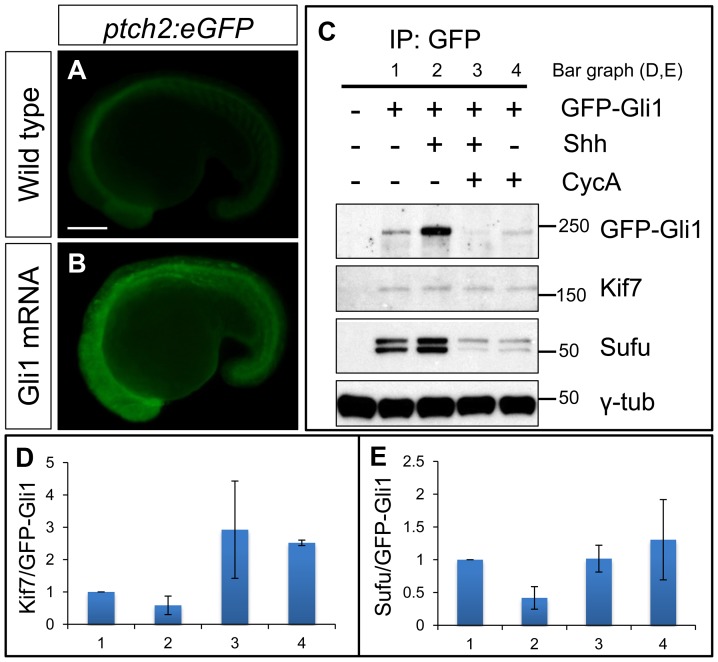
Functional tagged Gli1 associates with Kif7 and Sufu in a Hh dependent manner. (A,B) Lateral views of 20ss *ptc2:eGFP* embryos uninjected (A) and injected with *mCherry-Gli1* mRNA (B) showing the ectopic activation of *ptch2:eGFP* reporter. Scale bar: 150 µm. (C) Western blot analysis of anti-GFP immune-precipitates from uninjected embryos or embryos injected with a combination of GFP-Gli1 and/or Shh mRNA and/or exposed to cyclopamine (CycA); note the stabilization of GFP-Gli1 in the presence of ectopic Shh and a reduced association between Kif7 and Gli1 (see D for quantification); inhibition of Hh pathway activity by cyclopamine reverses this effect and further enhances the association. The inhibitory association of Sufu and Gli1 is also reduced in response to pathway activation by Shh mRNA injection (see E for quantification). (D) The ratio of Kif7:GFP-Gli1 from experiments described in panel (C); WT (1), Shh mRNA injected (2), CycA exposed and Shh mRNA injected (3), and CycA exposed embryos; showing reduced Kif7-Gli1 association upon pathway activation and increased association when the pathway is inhibited. Error bars represent standard deviation obtained from three independent biological replicates. (E) The ratio of Sufu:GFP-Gli1 from experiments described in panel (C); WT (1), Shh mRNA injected (2), CycA exposed and Shh mRNA injected (3), and CycA exposed embryos; showing reduced Sufu-Gli1 association upon pathway activation and restoration of this association upon pathway inhibition. Error bars represent standard deviation obtained from three independent biological replicates.

The genetic data imply that Kif7 may physically interact with Gli1. Since no antibody specific for zebrafish Gli1 is available, we tested this inference by expressing a GFP tagged form of Gli1 in wild-type embryos. Injection of mRNA encoding a similarly modified form of Gli1 tagged with mCherry resulted in ectopic activation of a *ptch2:eGFP* reporter gene (Fig. 5A,B), confirming that the tag does not disrupt the function of the protein. Immunoprecipitation of the eGFP-Gli1 from injected wild-type embryos revealed an interaction with both Kif7 and the negative Hh pathway regulator Sufu (Fig. 5C). The levels of Kif7 pulled down with eGFP-Gli1 were increased in embryos treated with cyclopamine (Fig. 5D), implying that the interaction is sensitive to Smo activation. Consistent with this, co-injection of Shh mRNA with the eGFP-Gli1 mRNA resulted in a decrease in the levels of Kif7 relative to Gli1 (Fig. 5D). A similar trend was also seen for the interaction between Sufu and Gli1 (Fig. 5E).

### Kif7 potentiates Gli2a activity by promoting its dissociation from Sufu

**Figure 6:**
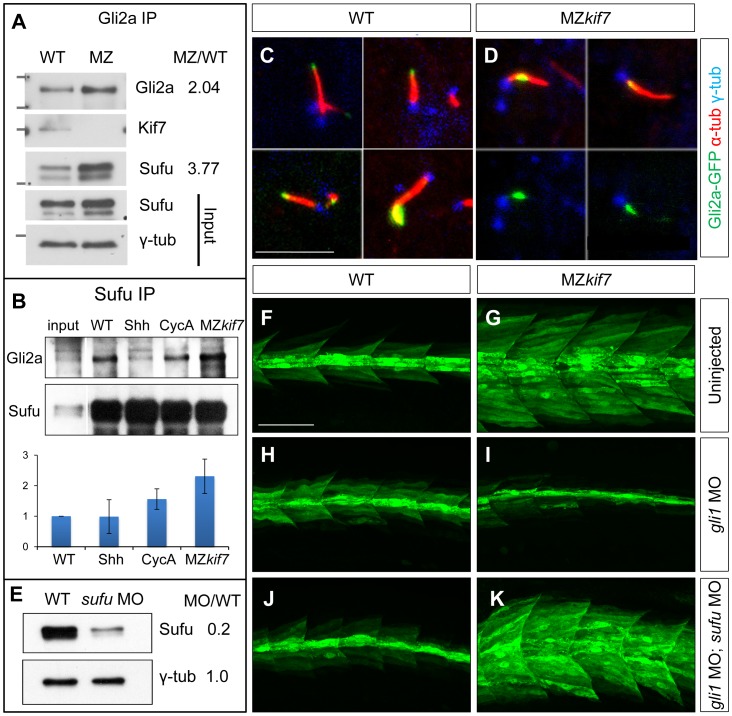
Kif7 modulates Gli2a localization and its association with Sufu. (A) Western blot analysis of anti-Gli2a immune-precipitates of WT and MZ*kif7* (MZ) embryos, showing association of Kif7 with Gli2a in wild-type embryos and increased association of Sufu with Gli2a in MZ*kif7* embryos, as indicated by the ratio of the normalized intensities of the MZ to WT signals. (B) Western blot analysis of anti-Sufu immuno-precipitates of WT, Shh RNA injected (Shh), cyclopamine (cycA) exposed and MZ*kif7* (MZ) embryos; the Gli2aFL:Sufu ratios normalized to wild-type are shown below each lane. Error bars represent standard deviation obtained from three independent biological replicates. Note the increase in levels of Gli2aFL that co-precipitates with Sufu in MZ*kif7* embryos. (C,D) Localization of a functional eGFP tagged Gli2a protein (green) to primary cilia in wild-type (C) and MZ*kif7* (D) embryos. The axonemes of the primary cilia are marked by acetylated α-tubulin (red) and the basal bodies by γ-tubulin (blue) staining. In wild-type, Gli2a localizes to the tip of the cilia whereas in MZ*kif7*, localization is restricted to the base of the cilia; the lower two panels in (D) are the same images as in the upper panels but with the red channel removed to show the juxtaposed GFP-Gli2a and γ-tubulin signals more clearly. Scale bar: 5 µm. (E) Western blot of wild-type (WT) and *sufu* morpholino-injected (s*ufu* MO) embryo extracts probed with anti-Sufu and anti γ-tubulin, showing significant depletion of Sufu levels in the morphants relative to wild-type (MO/WT). (F–K) Parasagittal optical sections of 30 hpf wild-type (WT) or MZ*kif7* embryos showing the effect on *eng2a:eGFP* expression of morpholino mediated knockdown of *gli1* (H,I) or *gli1* and *sufu* (J,K). Depletion of *gli1* in WT embryos (H) has no discernible effect, whereas it causes a drastic suppression of the ectopic expression in MZ*kif7* (I). This suppression is abrogated by simultaneous removal of Sufu and Gli1 from MZ*kif7* embryos (K). Scale bar: 50 µm.

In *Drosophila*, Cos2 exerts a positive effect on Ci activity by promoting its dissociation from Sufu [15] and in mammalian cells dissociation of Gli3 from Sufu has been shown to be of key importance for maximal pathway activation [25], [26]. We immunoprecipitated Gli2a from wild-type and MZ*kif7* embryos and used Western blotting to analyze its interaction with Sufu and Kif7. As expected, both Sufu and Kif7 proteins co-precipitated with Gli2a from wild-type embryos, whereas only Sufu could be detected in the immunoprecipitates from MZ*kif7* embryos (Fig. 6A). Notably, the levels of Sufu were increased approximately 2-fold relative to wild-type in the MZ*kif7* embryos, consistent with a decrease in dissociation of the two proteins in the absence of Kif7 function. Similarly, the levels of Gli2a that co-immunoprecipitated with Sufu from MZ*kif7* embryos were increased (2-fold) relative to wild-type controls (Fig. 6B); by contrast, activation of the pathway upstream of Kif7 through Shh mRNA injection, had no significant effect on the levels of Gli2a pulled down with Sufu, whilst a modest increase was seen in embryos treated with cyclopamine (Fig. 6B).

Dissociation of the Gli proteins from Sufu has been postulated to occur at the tip of the primary cilium [26]; in line with our findings, we found that the Gli2a-GFP fusion protein, which localizes to the tip of the primary cilium in wild-type embryos in response to Hh activity [38], was consistently located close to the base of the axoneme of primary cilia of myotomal cells in MZ*kif7* mutant embryos (Fig. 6C,D,S2).

Together, these data suggest a role for Kif7 in promoting the activation of full length Gli2a through its transport to the tip of primary cilium and dissociation from the Sufu protein. To test this inference, we used two previously characterized antisense morpholino oligonucleotides [30], [36] to reduce Sufu and Gli1 activity simultaneously in MZ*kif7* mutant embryos. Such embryos showed an 80% reduction in Sufu protein levels (Fig. 6E) and a significant restoration of the MZ*kif7* phenotype, with ectopic *eng2a:eGFP* expression throughout the fast-twitch fibers (cf. Fig. 6I & K). It follows that Kif7 not only potentiates Gli2a activity by promoting its dissociation from Sufu but also restrains it in a manner independent of its processing. Notably, knock-down of Sufu in MZ*kif7* mutant embryos resulted in a substantial increase in the number of slow twitch fibers and MP cells, implying a greater degree of Hh pathway de-repression than occurs in embryos lacking only Sufu or Kif7 (Fig. 7A–D,I). Consistent with this phenotype, these embryos showed increased expansion of the *ptch2* expression domain (Fig. 7E–H). This implies that Sufu also attenuates Gli1 activity and that Kif7 acts in concert with Sufu to restrain Gli1 activity in the myotome.

### Differing requirements for Kif7 in Hh pathway activity in the neurectoderm and the mesoderm

**Figure 8:**
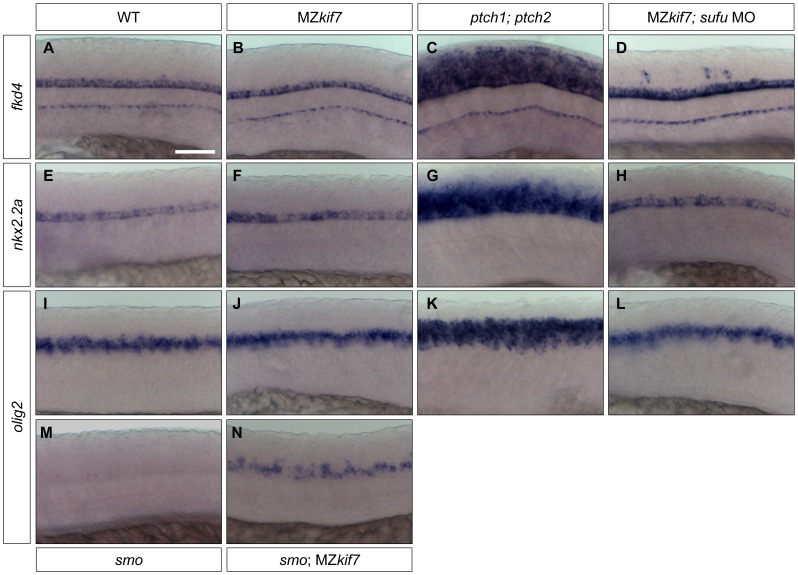
Hh target gene regulation in the neural tube is largely independent of Kif7 and Sufu function. (A–L) Lateral view at the level of yolk extension of 30 hpf WT (A,E,I), MZ*kif7* (B,F,J), *ptch1;ptch2* double mutants (C,G,K) and *sufu* MO injected MZ*kif7* mutants (D,H,L) hybridized with antisense probes for *fkd4* (A–D), *nkx2.2a* (E–H) or *olig2* (I–L); Note the ventrally restricted domains of *fkd4*, *olig2* and *nkx2.2a* typical of WT embryos are dramatically expanded in *ptch1;ptch2* double mutant embryos (C,G,K) but are unaffected in the absence of Kif7 function. *sufu* MO injected MZ*kif7* embryos show wild-type patterns of *nkx2.2a* or *olig2* but ectopically expression *fkd4* in scattered cells. (M,N) expression of *olig2* is completely lost from the neural tube in *smo* mutant embryos (M) but is partially restored by removal of *kif7* function (N). Scale bar: 50 µm.

Shh plays a major role in patterning the neural tube and the de-repression of the pathway caused by *ptch* mutations results in the ectopic expression of Hh target genes both in mouse [39], [40] and fish embryos [41], [42](Fig. 8C,G,K). In mouse embryos homozygous for loss of function *kif7* alleles, there is a modest expansion of the expression domains of *ptch1* and of the ventral neural tube markers *nkx2.2* and *olig2* [18], [19], [20] consistent with a partial de-repression of Hh pathway activity. In zebrafish MZ*kif7* embryos, by contrast, no changes in the neural tube expression domains of *fkd4*, *nkx2.2a* or *olig2* could be detected (Fig. 8B,F,J). Notably, however, we found that *olig2* expression could be partially uncoupled from its dependence on Smo activity by complete elimination of Kif7 activity (Fig. 8M,N). Taken together, these findings imply that the role of Kif7 in regulating Hh pathway activity is dispensable in the neural tube in zebrafish.

Given that Sufu knock down significantly enhances the MZ*kif7* phenotype in the myotome, we investigated whether Sufu activity might account for the lack of pathway de-repression in the neural tube in MZ*kif7* mutants. Surprisingly, knock down of Sufu function in MZ*kif7* embryos had no effect on the expression domains of either *nkx2.2a* or *olig2* (Fig. 8H,L); however, ectopic expression of *fkd* was consistently observed in the occasional cell scattered throughout the neural tube (Fig. 8D).

### *Drosophila* Cos2 can substitute for Kif7 in the zebrafish embryo

**Figure 9:**
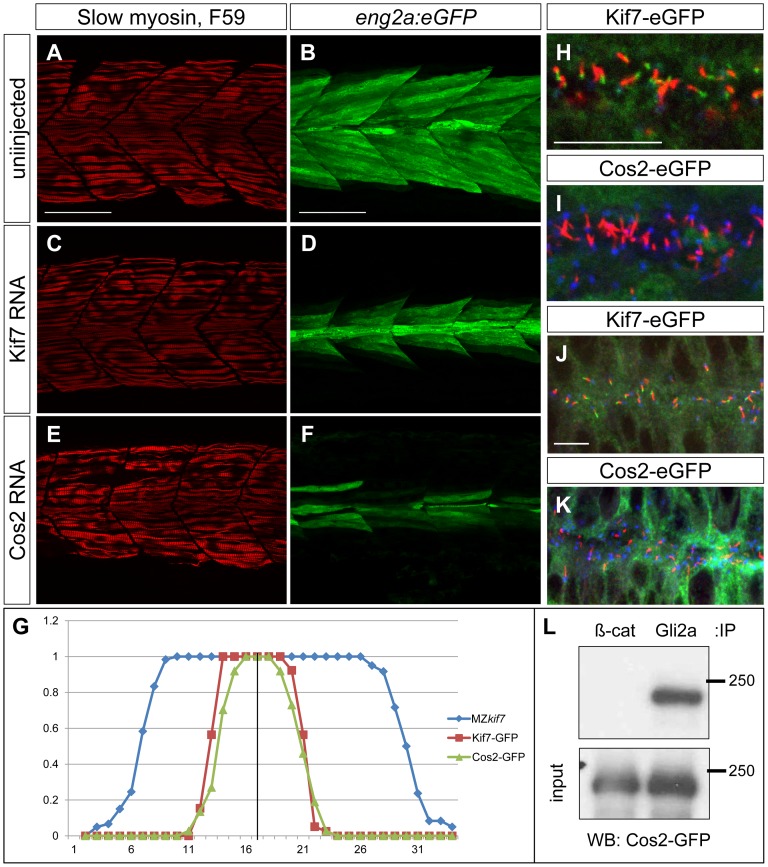
Drosophila Cos2 rescues Kif7 function and binds endogenous Gli2a. (A–F) Parasagittal optical sections of 30 hpf MZ*kif7*;*eng2a:eGFP* embryos showing slow twitch muscle fibers revealed by mAbF59 in red (A,C,E) and *eng2a* expression in green (B,D,F); control (A,B) embryo exhibits the typical expansion of GFP expression which is suppressed in embryos injected with zebrafish *kif7* mRNA (C,D) or *Drosophila cos2* mRNA (E,F). Scale bar: 50 µm. (G) Quantification of the *eng2a:eGFP* expression at 30 hpf in MZ*kif7* and in MZ*kif7* injected with mRNA encoding zebrafish Kif7 or *Drosophila* Cos2. Individual fibers (MFFs) were counted at the level of the yolk extension in 33–36 hemisegments from >10 embryos for each sample. Optical sections showing localization of zebrafish Kif7-eGFP (H,J) or *Drosophila* Cos2-GFP (I,K) in the otic vesicle (H,I) or neural tube (J,K) of 20ss embryo injected with mRNA encoding the tagged proteins: eGFP (green), α-acetylated tubulin (red) and γ-tubulin (red in H,J and blue in I,K). Scale bar 10 µm. (L) Western blot analysis of anti-β-catenin or anti-Gli2a immune precipitates from 20ss embryos injected with Cos2-eGFP RNA probed with anti-GFP revealing co-precipitation of the tagged Cos2 with endogenous Gli2a.

Sequence comparison has revealed significant structural conservation between *Drosophila* Cos2 and zebrafish Kif7 [17]. To investigate the extent to which function is also conserved, we exploited the ability to produce pure populations of MZ*kif7* embryos to assay the rescuing activity of *in vitro* synthesized mRNA encoding eGFP tagged forms of Cos2 and Kif7. Consistent with previous reports of mRNA mediated rescue of the *kif7* morphant phenotype [21], injection of zebrafish *kif7* mRNA into MZ*kif7* embryos resulted in substantial suppression of the ectopic *eng2a:eGFP* expression (Fig. 9A–D,G and data not shown). Remarkably, a significant degree of rescue was also seen following injection of *Drosophila cos2* mRNA (Fig. 9E,F). Confocal imaging of injected embryos revealed that the exogenous Kif7 protein localized to the tips of primary cilia (Fig. 9H,J) as previously reported [35]. By contrast, the tagged Cos2 protein was found exclusively in the cytoplasm, with no evidence of localization to the primary cilia (Fig. 9I,K). Immunoprecipitation of Gli2a from embryos injected with Cos2 mRNA revealed an interaction between the two proteins (Fig. 9L), consistent with the notion that Cos2 restrains the activity of Gli2a as well as Gli1 by retaining it in the cytoplasm.

### The stability and subcellular localization of Kif7 is regulated by Hh activity

**Figure 10:**
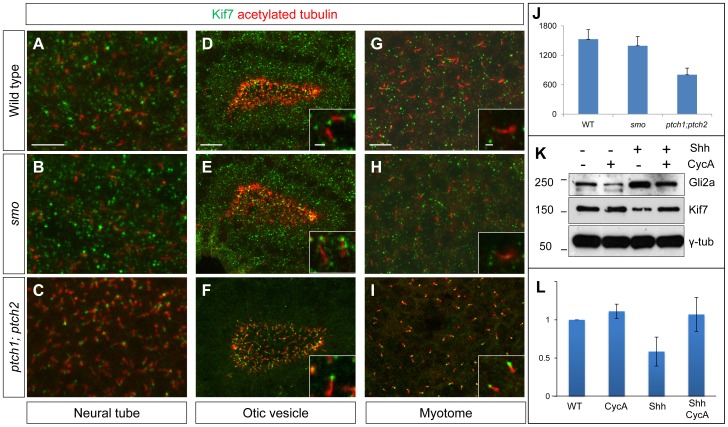
Kif7 protein localization is modulated by Hh pathway activity. (A–C) Parasagittal optical sections of the neural tube in 20ss embryos, showing the distribution of the endogenous Kif7 protein. In wild-type (A) and *smo* (B) embryos, Kif7 accumulates in puncta throughout the cytoplasm as well as in the primary cilium. In *ptch1; ptch2* double mutant embryos (C) by contrast, the cytoplasmic puncta are completely absent with Kif7 remaining only at the tips of the cilia. Scale bar: 10 µm. (D–I) similar distributions of Kif7 are seen in the otic vesicle (D–F) and the myotome (G–I) of wild-type and mutant embryos. Insets show a magnified view of a part of each image; note that Kif7 accumulates at the tips of some primary cilia in wild-type (D,G), *smo* mutant (E,H) but at elevated levels in all cilia in *ptch1;ptch2* double mutant (F,I) embryos. Scale bars: 10 µm; Inset scale bar: 1 µm. (J) Quantification of fluorescence intensity of Kif7 from the otic vesicle at 20ss from wild-type (WT), *smo* mutants and *ptch1;2* double mutants, revealing a decrease in Kif7 levels detected by immunofluorescence. Error bars represent standard deviation in spot intensity in pre-processed confocal stacks. (K) Western blot analysis of endogenous Gli2a and Kif7 protein from wild-type (WT), cyclopamine exposed wild-type (WT), Shh RNA injected (Shh) and cyclopamine treated Shh RNA injected (Shh CycA) wild-type embryos exposed to cyclopamine. Note the increase in full-length Gli2a levels following Shh overexpression. Relative levels of Kif7 protein normalized to wild-type are indicated in (L).

Using a rabbit polyclonal antibody raised against the C-terminal domain of zebrafish Kif7 (see Materials and Methods) we analyzed the sub-cellular distribution of the endogenous protein in fixed embryos. In wild-type embryos, Kif7 was found to accumulate in large puncta within the cytoplasm of cells in the neural tube, otic vesicle and somites; in addition, we found that it localized to the primary cilia of some cells, especially in the otic vesicle (Fig. 10A,D,G and data not shown). A similar distribution was observed in *smo* mutant embryos (Fig. 10B,E,H). By contrast, we found that the cytoplasmic puncta are completely absent in *ptch1;ptch2* double mutants, the protein localizing exclusively to the tips of primary cilia (Fig. 10C,F,I). We used spot segmentation and brightness measurement to quantify Kif7 protein levels in the otic vesicles (see Materials & Methods); this revealed an approximately two fold reduction in fluorescence intensity in *ptch1;ptch2* double mutants compared to *smo* mutants or wild-type siblings (Fig. 10J) suggesting that Hh pathway activity may induce increased turnover and/or dispersion of the Kif7 protein. Consistent with the former possibility, Western blot analysis of total extracts from embryos injected with Shh mRNA showed a similar two fold reduction in Kif7 levels relative to wild-type, an effect that could be reversed by exposure of the injected embryos to cyclopamine (Fig. 10K,L).

## Discussion

Through ZFN mediated targeted mutagenesis, we have confirmed a role for Kif7 in Hh pathway regulation in the zebrafish, previously inferred from transient knockdown experiments using morpholino antisense oligonucleotides [17]. Notably, we find that maternal expression of Kif7 is sufficient to support normal Hh pathway activity during embryogenesis, underlining the limitations of forward genetic screens for zygotic lethals in identifying genes with developmental functions.

Previous studies in mouse have revealed a role for mammalian Kif7 in promoting the processing of the Gli3 protein to its repressor form [18], [19], [20], analogous to the role of Cos2 in promoting cleavage of Ci in *Drosophila*. In cultured mammalian cells, tagged forms of Kif7 were found to localize to the base of primary cilia, translocating to their tips in response to Hh pathway activation [19], [20]. This led to the suggestion that Kif7 acts to localize Gli proteins to the base of the cilium, a site enriched in proteasomes, thereby promoting their proteolytic cleavage [20]. In this view, translocation of Kif7 to the cilia tips in response to Hh would abrogate Gli processing, leading to the activation of Hh target gene expression. Notably, however, we find that GFP tagged Gli2a remains close to the base of the primary cilium in MZ*kif7* mutants; moreover, the processing of endogenous Gli2a appears to be delayed rather than disrupted in the absence of Kif7 function. Thus although at 18 hpf the levels of the R form in MZ *kif7* embryos are reduced compared to wild-type, by 22 hpf the Gli2a FL∶R ratio is similar to that in wild-type embryos. Moreover, our genetic data indicate that Gli2a activity is largely dispensable for the ectopic activation of the Hh target genes in MZ*kif7* mutant embryos, whereas loss of Gli1 activity effectively suppresses their expression. This suggests that Gli1 is the major target of Kif7 repression; consistent with this, we find that GFP tagged Gli1 protein interacts with endogenous Kif7, an interaction that is attenuated by the activation of the Hh pathway. These findings contrast with the evidence from mouse studies implicating Gli2 as the principal Kif7 target [20]; notably, however, the activity of mammalian Gli1 expressed in transgenic *Drosophila* can be suppressed by endogenous Cos2 function [43]. We note that ectopic Gli1 activity has also been shown to underlie the Hh gain of function phenotypes of the zebrafish *igu* and *oval* mutants [44], [45], [46], in both of which the primary cilium is disrupted [38], [44], [47]. How this relates to our proposed role for Kif7 requires further investigation.

Although we found that endogenous Kif7 protein localizes to the primary cilia in the developing zebrafish embryo, we also detected significant accumulation of endogenous Kif7 protein outside the primary cilium, in cytoplasmic puncta. These puncta represent the major site of Kif7 accumulation in unstimulated cells, but disappear upon pathway activation. We surmise that this cytoplasmic pool of Kif7 plays a central role in the negative regulation of Gli proteins, an interpretation supported by our finding that Cos2, which functions independently of primary cilia in *Drosophila* and does not localize to primary cilia when expressed in zebrafish embryos, can nevertheless effect a substantial rescue of the MZ*kif7* phenotype. In this view, the Hh mediated dispersal of cytoplasmic Kif7 would facilitate pathway activation by releasing Gli proteins from their cytoplasmic sequestration, similar to the regulation of Cos2 by Hh in *Drosophila* [48]. Consistent with this, we find the interaction between Gli1 and Kif7 to be abrogated by Shh overexpression.

Translocation of Gli proteins to the tip of the primary cilium has been implicated in their activation by dissociation from Sufu [25], [26] and in line with the block in Gli2a translocation seen in MZkif7 embryos, we find a concomitant increase in the association of Gli2a with Sufu, similar to that recently reported in keratinocytes of mouse *Kif7* mutants [49]. This suggests that the principal role of Kif7 in the primary cilium is to mediate Gli protein activation by promoting its dissociation from Sufu. Consistent with this, ectopic Hh target gene activation is significantly enhanced by depletion of Sufu protein in MZ*kif7* mutant embryos. Such a role mirrors the promotion of dissociation of Sufu from Ci by Cos2 in *Drosophila*, an effect mediated by hyperactivation of the Fused serine threonine kinase in response to Cos2 dimerization [15]. In mouse, activity of the Fused orthologue Stk36 has been shown to be dispensable for Hh signaling [50] though in zebrafish, morpholino mediated knockdown experiment suggest that its involvement in the pathway has been conserved [30], [33]. Whether or not Stk36 is mediates the positive regulatory activity of Kif7 in zebrafish remains to be determined. Interestingly, we also observed accumulation of a faster mobility form of the FL Gli2a protein in MZ*kif7* embryos, similar to previous observations of Gli2 in *kif7* mutant mouse keratinocytes [49]. Moreover, this form accumulates at the expense of a slower migrating form that we found to be cyclopamine sensitive, suggesting that Kif7 is also required for a Smo-dependent modification of the FL form, a modification that may contribute to Gli2a activation.

It is remarkable that even in the total absence of Kif7 function, zebrafish can complete embryogenesis and survive to become fertile adults. This stands in contrast to the peri-natal lethality caused by loss of function alleles of *kif7* in the mouse [18], [19], [20] but interestingly, mirrors the finding that some Acrocallosal and Joubert syndrome patients are homozygous for loss of function alleles of the human *KIF7* gene [21], [31]. A further notable interspecies difference is the lack of requirement for Kif7 in the patterning of the neural tube in zebrafish. Despite this apparent dispensability, the Hh dependent regulation of Kif7 protein sub-cellular localization occurs within the neural tube and evidence of its activity can be detected in the absence of Smo function. We surmised that Kif7 might function redundantly with Sufu to repress Gli activity in the neural tube; however, despite causing a significant enhancement of the myotomal phenotype in MZ*kif7* mutants, morpholino mediated depletion of Sufu protein caused only sporadic ectopic expression of *fkd4*, a marker of floorplate, that by contrast is expressed throughout the neural tube in the absence of Ptch function. In these respects, the regulation of Gli activity by Hh signaling seems to differ significantly between mammals and zebrafish; in mouse the requirement for Sufu is absolute, its elimination causing almost total de-repression of pathway activity with consequent widespread defects in neural tube patterning similar to those caused by lack of Ptch1 function [51], [52]. Although Sufu is effectively dispensable in *Drosophila* [53], it seems surprising that Gli activity can be restrained in the absence of both Sufu and Kif7 in the zebrafish neural tube; one caveat to this conclusion, however, is that the morpholino mediated depletion of Sufu is not complete. Nevertheless, it seems clear that some other component(s) must be responsible for the suppression of Gli activity. In the absence of Kif27 [17], [33] or any other close homologue of Kif7 from the zebrafish genome, the identity of such components remains a mystery.

## Materials and Methods

### Ethics statement

The research described in this paper uses the zebrafish as an alternative to mammalian experimental models. Adult zebrafish were raised and maintained under internationally accepted conditions. All experimental procedures were performed in compliance with and approved by the A*STAR Biological Resource Centre Institutional Animal Care and Use Committee (IACUC Project #110638). Most experimentation and analysis was restricted to the first five days post fertilization (dpf). Homozygous mutant fish were regularly monitored and any showing signs of distress were humanely euthanized following accepted protocols.

### Zebrafish strains and husbandry

Adult fish were maintained on a 14 hour light/10 hour dark cycle at 28°C in the AVA (Singapore) certificated IMCB Zebrafish Facility. Previously described zebrafish strains used were: *ptch1^hu1602^*, *ptch2^tj222^* [41]; *igu^ts294^* [46]; *Tg(eng2a:eGFP)^i233^* [54]; *gli1 (dtr^ts269^)* [55]; *gli2a (yot^ty119^*) [56] *gli2a^i276^* [37]; *smo^b641^* [57].

### DNA expression constructs and zebrafish transgenic lines

Ptch2:HAeGFP: Homologous recombination in bacteria [58] was used to insert eGFP-FRT-AMP-FRT in frame after the first codon of *ptch2* in BAC CH211-226H23. An HA tag was inserted in frame at the N-terminus of eGFP, while amplifying the targeting cassette. The FRT-Amp-FRT was excised using arabinose induction and a fragment containing 8 kb upstream of the insertion along with eGFP was cloned into pMiniTol2 using homologous recombination for gap repair [58]. The targeting cassettes and plasmids used have been described previously [54]. More than 10 stable transgenic lines were generated with this construct and one of the brightest of these (allele designation *i271*) selected for further experiments.

pCS2-mCherry-Gli1, pCS2-eGFP-Gli1: A Vn-Gli1-pCS2+ plasmid [54] was digested with SmaI and AgeI (to release the Vn fragment); eGFP and mCherry were amplified with oligos containing the SmaI and AgeI and cloned into the same site of Vn-Gli1-pCS2+ plasmid.

pCS2-Kif7-eGFP, pCS2-Cos2-eGFP: Zebrafish Kif7 and *Drosophila* Cos2 [10] were amplified using BamHI and SmaI adaptors and cloned into the same sites of a eGFP-pCS2+ plasmid (eGFP cloned between SmaI, XbaI sites)

### Generation, selection and genotyping of *kif7* mutant alleles

Plasmids encoding Zinc-finger nucleases (ZFN) specific for the zebrafish *kif7* (set 3) gene were purchased from Sigma (see accompanying data sheet). Capped polyadenylated RNA from each plasmid was produced by *in vitro* transcription and a range of doses was injected into one-cell stage zebrafish embryos. Embryos injected with approximately 600 pg had around 30% rate of deformity at 24 hpf (hours post-fertilization). Genomic DNA prepared from non-deformed 24 hpf embryos injected with this dose was used as a PCR template to analyze potential somatic mutations. Roche Titanium 454 amplicon sequencing showed that 2.5% (6.5% is by 8 of 127 colony PCR and sequencing) of the amplicon molecules had insertions or deletions at the target site. G0 adults derived from embryos injected with ZFN capped RNA were in-crossed and their progenies (G1) individually genotyped by PCR using the forward primer (Kif7 exF2: 5′CGAGGTGCTGAGTCTCTTAGAGT) and reverse primer (Kif7 exR1: 5′TGAATCCCTGTATGGGATATGGGT) followed by Sanger sequencing.

Founders transmitting two different alleles (kif7Δ8, an 8-bp del: GGACCTGG kif7Δ7, a 7-bp del: TTGTGGA) were selected and used to establish stable mutant lines. The full nucleic acid sequences of mutated alleles together with their allele designations are shown in Table 1).

### *In situ* hybridization and immunofluorescence

Standard *in situ* hybridization (ISH) was performed with anti-DIG alkaline phosphatase and chromogenic substrate NBT/BCIP as previously described [59]. RNA probes were prepared from templates as previously described: *nkx2.2* [60], *olig2* [61], *ptch2* (formerly *ptc1*) [62], *prdm1a* [63].

Whole-mount antibody staining was performed as previously described [35], [64] at the following dilutions: mAb 4D9 (anti-Engrailed; DHSB) at 1∶50–1∶200; mAb F310 (1∶50; DHSB); mAb F59 (1∶50; DHSB); rabbit anti-Prox1 (1∶2000); rabbit anti-γ-tubulin (1∶500; Sigma); mouse anti-γ-tubulin (1∶500; Sigma); mouse anti-acetylated α-tubulin (1∶800; Sigma); rabbit anti-Kif7 (1∶500); chick anti-GFP (1∶400; Abcam); rabbit anti-GFP-488 (1∶750; Invitrogen); rabbit anti-DSred (1∶200; Clontech). The secondary antibodies were: Alexa488-conjugated goat anti-mouse or rabbit, Alexa546-conjugated goat anti-mouse and Alexa568-conjugated goat anti-rabbit, Alexa633-conjugated goat anti-secondary antibodies (1∶1000, Invitrogen). Bright field microscopy images were acquired with an AxioCam HRc mounted on a Zeiss AXIO Imager M2, Olympus DP70 on MVX10 or Leica DFC300 FX mounted on MZ16FA. Fluorescent specimens were imaged using the 60× or 100× oil immersion objective on an Olympus Fluoview 1000 confocal microscope. Images were acquired using Olympus FV10-ASW software.

### Synthetic RNA, DNA and morpholino for injection

Capped synthetic mRNAs for the following genes was synthesized using the SP6 mMessage mMachine Kit (Ambion), the plasmids were linearized with restriction enzymes as indicated: pCS2-GFP-Kif7 (NotI) [17]; pCS2-Cos2-GFP (NotI) [65]; pCS2-KIF7-GFP (NotI). BAC Gli2a-GFP was used as previously described [38]. Morpholino oligonucleotides were obtained from Gene Tools (USA) and injected into newly fertilized embryos. The sequences of those targeting Gli2a and Sufu antisense were as described [30]. The sequence of the Gli1 morpholino was as described by [36].

### Cyclopamine treatment of embryos

Cyclopamine treatment followed a standard method with immersion in 40 µM cyclopamine (Toronto Research Chemicals) from 50% epiboly as previously described [30].

### Generation of antibodies

Sufu: The full-length zebrafish Sufu coding sequence [30] was cloned into the BamHI-EcoR1 sites of pGEX-6p-1 (Amersham). The resulting GST-zSufu fusion protein was expressed in *E.coli* strain BL21 and purified by SDS-PAGE and electro-elution. After dialysis in PBS, the antigen was injected into mice. Splenocytes were extracted from immunoreactive animals and fused with melanoma cells to generate hybridomas. Of 904 hybridoma clones screened, 50 were positive by ELISA and one of these, 2A10, tested positive by western blot of zebrafish embryo extracts. Polyclonal rabbit anti-zebrafish Sufu antibody was generated by Absea Biotechnology Ltd. (China) using the same pGEX-zSufu expression construct.

Kif7: Polyclonal rabbit anti-zKif7 antibody was generated by Strategic Diagnostics Inc. (USA) using a peptide corresponding to residues 1231–1330 of the zebrafish Kif7 protein sequence [17].

### Fluorescence image analysis

#### (1) Image pre-processing

Acquired image stacks were reconstructed and the voxel size normalized using linear interpolation such that the scales of voxels were isotropic, *i.e.* each voxel is 0.2×0.2×0.2 µm. The images were smoothened by a Gaussian kernel to reduce noise and enhanced based on the histogram. A Laplacian kernel was applied to enhance the boundaries between them.

#### (2) Spot segmentation and brightness measurement

To formalize the spot segmentation problem, the pre-processed image stacks at each time point were defined on a subset of three-dimensional space 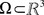. We used 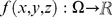 to represent the image intensity of the spots after the pre-processing. The intensity function of the image stack was normalized such that 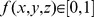. The local maximum in 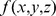 was detected as spot centers, denoted by seeds. The spots were then segmented by Evolving Generalized Voronoi Diagrams approach [66], [67]. The segmented regions of 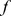 represent spot segments, represented by 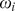 for 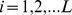, where *L* is the number of detected spots. Each 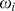 forms connected regions in 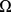. A simple statement defines the connected region:

**Connected region**: A set of points 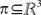 form a connected region if 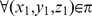 and 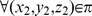, 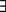 a path 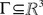 connecting 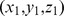 and 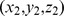 such that 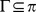.

The average brightness of each spot is then calculated based on Eq. (1),
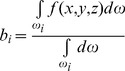
(1)where the denominator is the volume of spots.

### Western blot analysis

Embryos were de-chorionated, de-yolked, and homogenized manually in ice cold PBS without Ca2+ and Mg2+ in the presence of complete protease inhibitor cocktail (Roche). The embryo pellet was lysed in RIPA buffer (50 mM Tris.HCl, pH 8.0/150 mM NaCl/1%NP-40/0.5% Na.Deoxycholate/0.1%SDS/protease inhibitor cocktail/1 mM PMSF). Samples were microcentrifuged for 10 min at 4°C, loading buffer (62.6 mM Tris HCl, pH 6.8; 2% SDS; 0.01% bromophenol blue; 10% glycerol; 100 mM DTT) was added to the supernatant and the equivalent of 30 embryos run on each lane of a 7.5% acrylamide denaturing gel at 30 mA for 120 mins, and electroblotted onto Immobilon-P polyvinylidene fluoride (PVDF) membrane (Millipore). PVDF strips were blocked in 5% milk powder PBS 0.1%Tween20 for 1 hr, and incubated with rabbit anti-zebrafish Gli2a (1∶5000) [54], rabbit anti-zebrafish Kif7 (1∶5000), mouse anti-Sufu (1∶100) for 1 hr at room temperature. After washing, primary antibody was detected with ECL HRP-conjugated anti-rabbit lgG (1∶50,000) and anti-mouse IgG (1∶50,000). Chemiluminescent Substrate was SuperSignal West Femto (Pierce). The loading amount of protein extract among specimens was evaluated by gamma-Tubulin level with either rabbit or mouse anti-gamma Tubulin (1∶5000; Sigma). Signal quantification was performed using Adobe Photoshop or Image J software.

### Immunoprecipitation

Embryos were de-chorionated using pronase (2 mg/ml, Sigma) and rinsed with egg water and then PBS with 2× protease inhibitor (PI; Roche). Embryos were de-yolked by yellow tip on ice and rinsed in ice-cold PBS/2× PI. The embryos were centrifuged at 200×g for 5 mins. The pellet was stored at −80C degree. For 1000× embryos, 1 ml of triton buffer (20 mM Tris-HCl, pH 8.0/137 mM NaCl/10% Glycerol/1%Triton X100/2 mM EDTA)/2×PI/10 mM PMSF is added to extract the proteins. Tubes were left horizontally on ice for 30 mins or longer. The lysates were spun at 10,000×g for 15 mins. The suspension was transferred into a new 2 ml eppendorf. Forty µl of lysate was taken out as the input control. In 1 ml of lysate, 150 µl of magnetic Dyna-bead (Invitrogen) was added to pre-clear for 3 hours at 4°C degree. After removing the pre-clear beads, 10 µg of rabbit primary Ab was added into lysate. For eGFP immunoprecipitations we added the lysate to 20 µl of GFP-Trap beads (Chromotek) and other steps remained unchanged. The binding between rAb and the specific protein was facilitated by shaking at 4°C overnight. The 120 µl of dynabead protein A was added in 1 ml of lysate and incubated at 4°C degree for no more than 1 hour to pull down the complex of rAb-target protein. The beads were washed with 1.5 ml of lysis buffer/2×PI per tube 4 to 6 times. During the last time of washing, the beads were transferred to a new tube. Elution was performed by adding 40 µl of 2× SDS NuPage LDS/100 mM DTT (Invitrogen) per tube and by heating at 95°C for 5 mins.

## Supporting Information

Figure S1 Complete loss of Kif7 does not result in left-right patterning defects. Dorsal view of five 22ss MZ*kif7* embryos stained with an antisense probe for *lefty2* showing correct positioning of the heart tube. Up to 30 MZ*kif7* embryos were analyzed for lefty2 expression, all of which displayed the correct L-R patterning.
(TIF)

Figure S2 Localization of Gli2a-GFP in the primary cilia of wild-type (WT) and MZ*kif7* embryos. The graph shows the frequency of localization of Gli2a-GFP to different regions of the primary cilia in transient transgenic embryos.
(TIF)
