# Supplementary figures and images for: Positive and Negative Regulation of Gli Activity by Kif7 in the Zebrafish Embryo
Source: PLoS Genet. 2013 Dec 5;9(12):e1003955. doi: 10.1371/journal.pgen.1003955 (PMC3854788; doi:10.1371/journal.pgen.1003955)

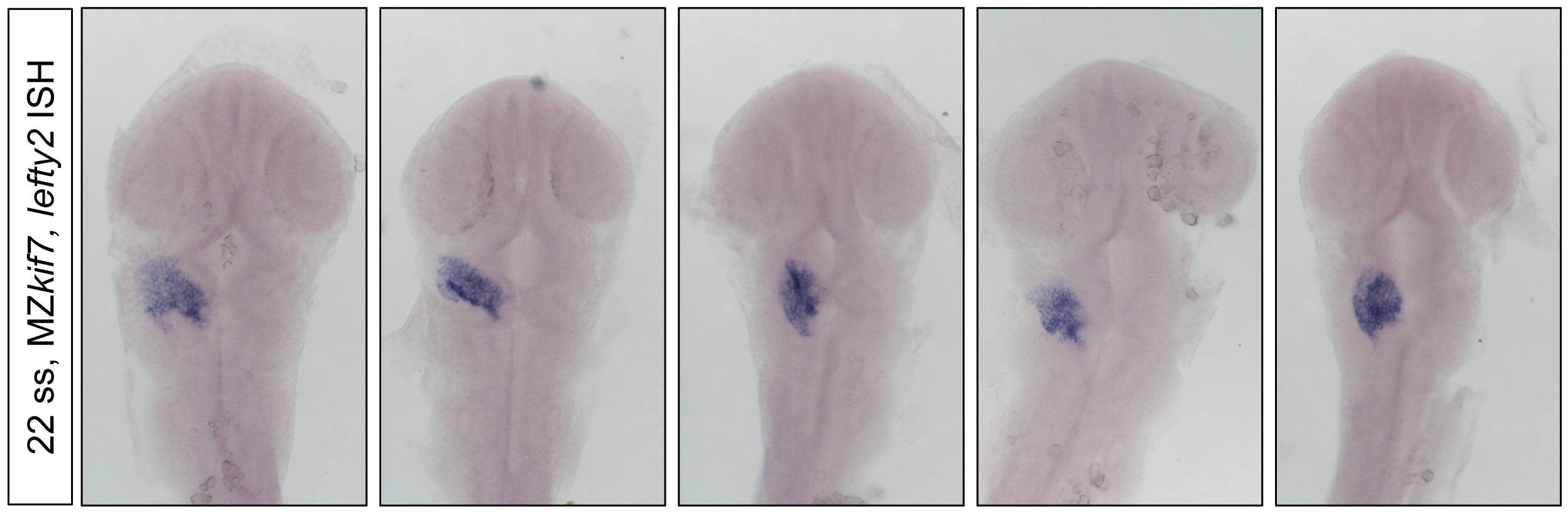

Supplement: Figure S1 — Complete loss of Kif7 does not result in left-right patterning defects. Dorsal view of five 22ss MZkif7 embryos stained with an antisense probe for lefty2 showing correct positioning of the heart tube. Up to 30 MZkif7 embryos were analyzed for lefty2 expression, all of which displayed the correct L-R patterning. (TIF) [file pgen.1003955.s001.tif]

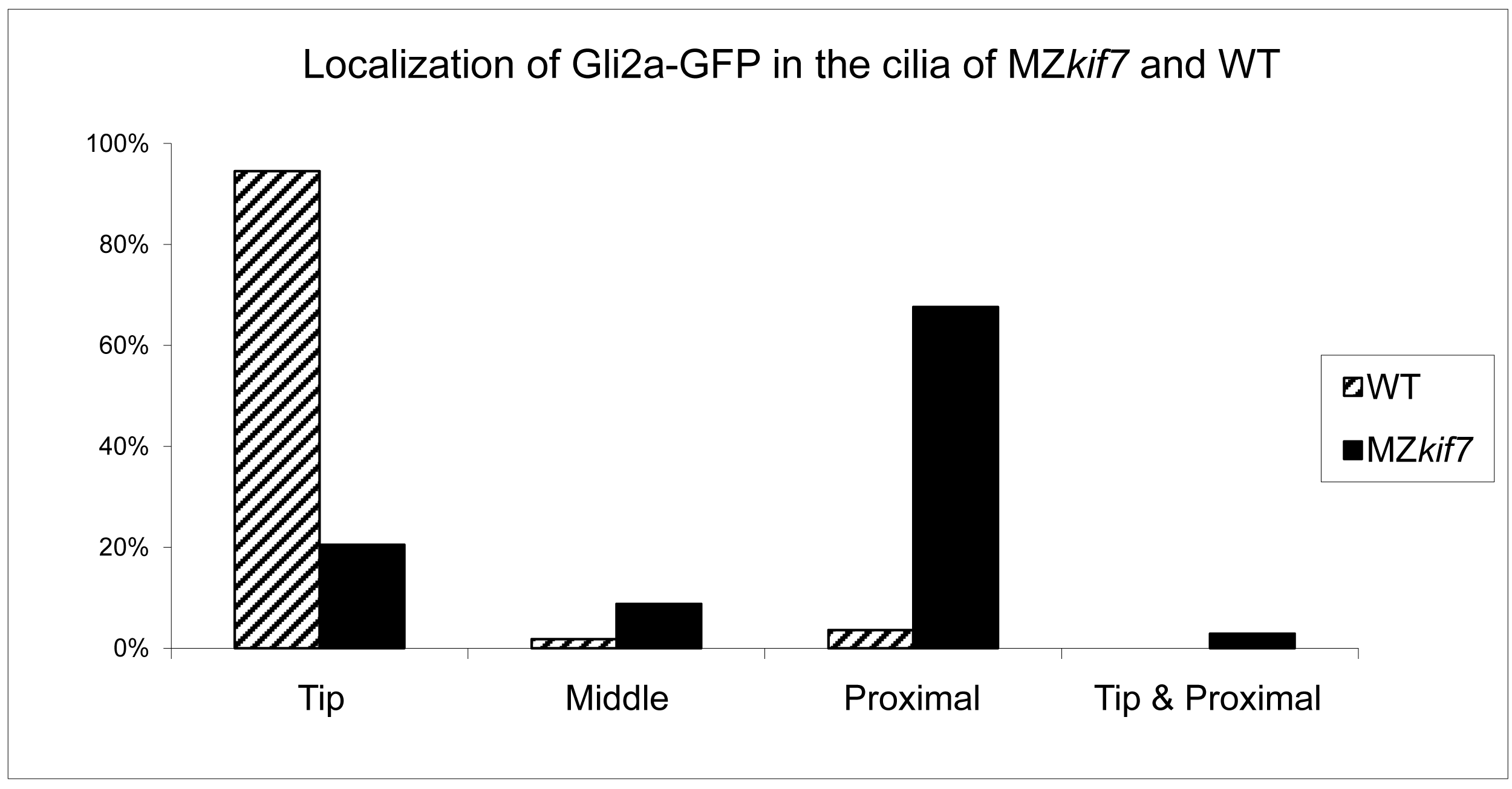

Supplement: Figure S2 — Localization of Gli2a-GFP in the primary cilia of wild-type (WT) and MZkif7 embryos. The graph shows the frequency of localization of Gli2a-GFP to different regions of the primary cilia in transient transgenic embryos. (TIF) [file pgen.1003955.s002.tif]
